# Masturbation Among Malaysian Young Adults: Associated Sexual and Psychological Well-Being Outcomes

**DOI:** 10.1007/s12119-023-10101-2

**Published:** 2023-05-26

**Authors:** Li Ann Phuah, Jaclyn Hui Jie Teng, Pei Hwa Goh

**Affiliations:** 1 Department of Psychology, Jeffrey Cheah School of Medicine & Health Sciences, Monash University Malaysia, 47500 Bandar Sunway, Subang Jaya, Selangor Malaysia; 2 School of Medical and Life Sciences, Sunway University, Sunway, Malaysia

**Keywords:** Masturbation, Self-stimulation, Sexual satisfaction, Culture, Gender differences

## Abstract

Despite being a common sexual practice, not much is known about masturbation within the Malaysian context. The current study examined the masturbation practices of a convenience sample of Malaysian young adults, and how one’s reported experience of masturbating and frequency of masturbation is associated with sexual and psychological well-being. Satisfaction with sex life, satisfaction with life, depression, anxiety, and stress were measured as outcome variables. Responses from 621 participants (*M* = 22.1 years, *SD* = 2.4 years) that were collected through an online questionnaire were analyzed. Results revealed that most participants (77.7%) had masturbated at least once in their life. Sexual satisfaction and psychological well-being did not differ between those who reported masturbating before and those who did not. Among those with masturbation experience, higher frequency of masturbation was associated with less sexual satisfaction and more symptoms of anxiety and stress. Masturbation frequency was not associated with life satisfaction or reports of depressive symptoms. Gender, frequency of partnered sex, availability of a sexual partner, and religiosity did not moderate the relationships between masturbation frequency with sexual satisfaction and psychological well-being. Although the limitations of the current study impede generalizability, masturbation was found to be a common behavior among the current sample. Causal inferences cannot be drawn from the significant associations found in the current study, as the findings indicate such relationships may be bidirectional.

**Supplementary Information:**

The online version contains supplementary material available at 10.1007/s12119-023-10101-2.

## Introduction

Masturbation, or self-stimulation for sexual pleasure, is a behavior that is an important part of sexual development (Coleman, 2003; Kaestle & Allen, 2011), which can be performed using one’s own hands, fingers, external objects or sex toys. Masturbation has been shown to improve people’s bodily knowledge or anatomical understanding of their bodies, cultivate positive feelings about their bodies (Shulman & Horne, 2003), and help people better understand what makes them feel good sexually (Bowman, 2014; Coleman, 2003). Indeed, masturbation is commonly encouraged in sex therapy as a means to help clients with sexual dysfunction increase self-knowledge about their sexual responses (Coleman, 2003). However, most findings on the benefits of masturbatory practices have been derived from North American populations (e.g., Regnerus et al., 2017; Rowland et al., 2020). Little is known about how this applies in a more sexually conservative culture.

Malaysia is a multicultural Southeast Asian country with a predominantly Muslim (61.3%) and Buddhist (19.8%) population (Department of Statistics Malaysia, 2010), where masturbation, and sexuality in general, remain taboo. Malaysians are generally discouraged from openly discussing sex, have more negative attitudes towards masturbation, and have little knowledge of sex and reproductive health (Sidi et al., 2013). This is unsurprising given that masturbation is considered unlawful in Islam (Hoseini, 2017) and believed to cause loss of *yang* or vital energy among men according to traditional Chinese medicine (So & Cheung, 2005). Hitherto, research on sexuality in Malaysia remains scarce. In the current study, we examined the masturbatory practices of a sample of young adults in Malaysia, and the sexual and psychological well-being correlates of masturbation. Although masturbation is a form of sexual behavior that can be done solitarily or in the presence of others, the current study examined masturbation within the context of solo sex and not in the context of partnered sex.

### Masturbation and Sexual Satisfaction

Masturbation, as a form of sexual behavior, has been found to contribute to one’s general sexual well-being. In interviews with 20 young women, Hogarth and Ingham (2009) found that participants’ positive or negative experiences with masturbation were consistently related to other areas of their sexuality (e.g., sense of control during sexual situations, condom use, and sexual awareness). Similarly, Hurlbert and Whittaker (1991) found that female participants in their quantitative study who were masturbators reported greater sexual satisfaction in their marriages than non-masturbators. While these studies indicate positive outcomes of masturbation for women, some past findings have revealed reduced sexual well-being among men who masturbate more frequently, notably greater sexual boredom (Carvalheira et al., 2015) and lower orgasm satisfaction in partnered sexual relationships (Cervilla & Sierra, 2022). On the one hand, individuals who masturbate more frequently may be more attuned to their sexual needs and feelings (Hogarth & Ingham, 2009; Hurlbert & Whittaker, 1991), be more confident in expressing themselves sexually, and thus cultivate more sexually rewarding relationships (Saliares et al., 2017) or experiences. However, other factors such as pornography use during masturbation (Miller et al., 2019) or poor relationship intimacy (Carvalheira et al., 2015) may act as deterrents to achieving positive sexual well-being in the context of partnered sexual relationships from masturbation. Taken together, we argue that masturbatory behaviors may help increase sexual satisfaction, which we define as general satisfaction towards all aspects of one’s sex life.

### Masturbation and Psychological Well-Being

Masturbation practices have also been found to be associated with psychological well-being. According to Burri and Carvalheira (2019), two commonly cited reasons to masturbate in their sample of German women were to cope with stress and also to relax. Oxytocin, a hormone associated with orgasm and various sexual activities, can bring about feelings of relaxation that aid in coping with stress (Magon & Kalra, 2011). Given that people who masturbate are more sexually aware and may be able to orgasm more effectively, it is reasonable to expect higher masturbation frequency to be associated with lower stress.

Although masturbation has been linked to lowering stress, Rowland et al. (2020) found that higher frequency of masturbation was also associated with higher levels of general anxiety or depression among women. However, findings on the associations between masturbation with anxiety and depression are scarce and mixed in the literature. While findings by Rowland et al. (2020) indicate a positive association, Jiao et al. (2019) suggest that masturbation may be associated with symptoms of anxiety and not depression. Masturbation has also been found to be inversely associated with life satisfaction (Brody & Costa, 2009; Långström & Hanson, 2006). Nonetheless, the relationship between masturbation and life satisfaction has not been studied extensively in the literature.

Considering the more sexually restrictive culture in Malaysia (Sidi et al., 2013), we argue that people who masturbate are more likely to report poorer psychological well-being (i.e., higher levels of anxiety and depression and lower levels of life satisfaction) than those who do not masturbate, possibly due to accompanying feelings of shame and guilt over the act. Zimmer and Imhoff (2020) stated that religious and political beliefs may contribute to feelings of shame and guilt with masturbation, which may result in abstinence from the behavior altogether. Although research has not established the link between masturbation and guilt specifically among conservative people, it has been found that people who have more conservative attitudes tend to masturbate less than people who hold more liberal social attitudes (Hatemi et al., 2017). Likewise, people who have never engaged in masturbation are more likely to be more conservative and uphold traditional sexual attitudes than those who have (DeLamater & Friedrich, 2002; Miller & Lief, 1976).

### Potential Moderators

The current study also examined the role of potential moderators in the associations between masturbation with sexual satisfaction and psychological well-being. While there remains a general stigma surrounding the topic of masturbation, a double standard exists whereby pleasure-seeking is generally viewed more favorably among men than women (Kaestle & Allen, 2011). Given the link between sexuality and shame and/or guilt among women (Carvalheira & Leal, 2013; Fahs & Frank, 2014), we expected more frequent masturbation to be associated with lower satisfaction with one’s sex life and psychological well-being among women and not men.

Taking into account the complementary and compensatory model of masturbation (Regnerus et al., 2017), we considered the role of sex frequency as a moderator for the relationship between masturbation with sexual satisfaction. The compensatory model posits that masturbation is used as an alternative or a substitute when partnered sexual activity is not feasible (Dekker & Schmidt, 2003), while the complementary model postulates that the increase or decrease in partnered sexual activity is believed to stimulate or diminish respectively the overall desire for sexual activity, which includes masturbation (Regnerus et al., 2017). Research has revealed mixed results, providing support for both models (Das et al., 2009; Driemeyer et al., 2017). Considering that the association between masturbation and partnered sexual activity may be less straightforward than theorized by the two models of masturbation, we examined frequency of partnered sex as a potential moderator of the association between masturbation and sexual satisfaction.

In addition to the frequency of partnered sex, we also take interest in relationship status as a potential moderator, as stable access to a sexual partner may indicate familiarity with each other’s sexual preferences. Partnered status, or the availability or access to a sexual partner, has been shown to be a good predictor of masturbation within the past two weeks (Regnerus et al., 2017). Research has found that masturbation is negatively associated with relationship intimacy and relational satisfaction (Carvalheira et al., 2015; Perry, 2019), which may contribute to reduced psychological well-being among those who are in a relationship. However, past studies have found that individuals in committed, exclusive relationships with their sexual partners were more likely to have higher sexual satisfaction (Higgins et al., 2011). Thus, accessibility to a sexual partner may enhance the positive relationship between masturbation and sexual satisfaction as well as the negative relationship between masturbation and psychological well-being.

Furthermore, the relationship between masturbation and its outcomes may not be as clear-cut considering the study’s Malaysian context, in which religious values and Asian cultures inform many sexual attitudes and behaviors (Stivens, 2006). As masturbation is generally viewed as a shameful and deviant sexual act by religious beliefs (Jiao et al., 2019), the negative belief or the cognitive component of masturbation may overtake the pleasure component, especially among more religious individuals. Indeed, guilt associated with masturbation has been found to be detrimental to psychological well-being (Jiao et al., 2019). As religiosity may weaken the benefits and enhance the drawbacks of masturbation, we also examined religiosity as a potential moderator for the relationship between masturbation with sexual and psychological well-being.

### The Current Study

To the best of our knowledge, this is the first study to investigate masturbatory behaviors among a sample of Malaysian young adults. The current study aimed to examine how masturbation was associated with general sexual satisfaction and psychological well-being, namely life satisfaction, depression, anxiety, and stress. We also examined the role of gender, frequency of sex, partner availability, and religiosity as moderators of these associations.

Based on theoretical and empirical evidence, we hypothesized that:

#### H_1_

Individuals who masturbate would have significantly higher levels of sexual satisfaction, depression, and anxiety as well as significantly lower levels of life satisfaction and stress than those who do not masturbate.

Additionally, we expected that gender, frequency of sex, partner availability, and religiosity would moderate the relationships between masturbation frequency with sexual satisfaction and psychological well-being. Specifically, we hypothesized that:

#### H_2a_

The association between masturbation frequency and sexual satisfaction would be negative among women and positive among men. In contrast, the relationship between masturbation frequency and psychological well-being would be positive among men and negative among women.

#### H_2b_

Higher frequency of partnered sex would strengthen the positive relationship between masturbation frequency and sexual satisfaction.

#### H_2c_

Having an available partner would strengthen the positive relationship between masturbation frequency and sexual satisfaction as well as between masturbation frequency with depression and anxiety, strengthen the negative relationship between masturbation and life satisfaction, and weaken the negative relationship between masturbation and stress.

#### H_2d_

Higher levels of religiosity would weaken the positive relationship between masturbation frequency and sexual satisfaction, strengthen the positive relationship between masturbation frequency with depression and anxiety, strengthen the negative relationship between masturbation and life satisfaction, as well as weaken the negative relationship between masturbation and stress.

## Methods

### Participants

Participants were Malaysian young adults aged 18 to 30 (*M* = 22.1, *SD* = 2.4) recruited through convenience and snowball sampling methods. Of the 780 recorded responses, 621 participants remained after excluding duplicates and responses that were more than 40% incomplete. Of these, most identified as female (60.5%; 39.5% male), Malaysian Chinese (58.8%; 18.4% Malay, 15.1% Indian, 7.7% others), and available to date (58.3%; 41.7% in exclusive relationships). Two participants did not provide their age and relationship status.

### Procedure

This study was part of a larger research project examining sexuality and well-being among Malaysian young adults, which was done in two phases. In the first phase of data collection (June to August 2019), recruitment was done using a face-to-face approach on campus, posts on social media, and snowballing, while the second phase (May to July 2020) relied on the distribution of flyers via social media, personal contacts of research assistants, and snowballing. All measures used in this study were available in both phases except for attitudes towards masturbation, pornography consumption during masturbation, and reasons for masturbating, which were only measured in the second phase of data collection.

In both phases of recruitment, participants were given an explanatory statement detailing the aims of the research project, the right to withdraw at any point, and any risks involved in participation. Those interested in participating provided their contact details to receive the link to the online questionnaire. In the explanatory statement, invitation message, and at the end of the questionnaire, participants were informed that the completion of the questionnaire implied consent. Upon completion, participants were debriefed, given an honorarium of MYR30.00 (approximately USD7.30) for their participation, and asked to sign a document that acknowledged their participation in the study and the receipt of the honorarium. This research was approved by the university’s Human Research Ethics Committee.

### Measures

#### Masturbation

Before participants answered measures related to their sexual history and attitudes, we provided a definition of sexual activity which includes sensual caressing, foreplay, masturbation, oral sex, and penetrative sex. For masturbation, we defined the behavior as “the act of touching oneself in a sexual manner while alone”. We also provided participants with some examples in the local slang, such as “play with yourself, touch yourself, ta fei kei, and toceng”.

##### Frequency

Participants rated their frequency of masturbation using a single-item measure that was rated on an 8-point Likert scale ranging from 1 (*never*) to 8 (*multiple times a day*).

##### Enjoyment

Participants rated their enjoyment of masturbation using a single-item measure that was rated on a 7-point Likert scale ranging from 1 (*extremely unenjoyable*) to 7 (ex*tremely enjoyable*).

##### Orgasm Frequency and Latency

Participants rated the frequency at which they achieve orgasm from self-stimulation on a 7-point Likert scale ranging from 1 (*never*) to 7 (*always*). Participants were also asked to state how long it typically takes them (in minutes) to orgasm during masturbation.

##### Age of First Masturbation

Participants were asked to state the age at which they had their first solo sexual experience (i.e., masturbation). Among those who have masturbated, 13 participants stated that they did not remember the age at which they first masturbated and were thus considered missing values.

##### Attitudes Towards Masturbation

Participants were asked to complete three statements regarding their general beliefs about masturbation. The options given were based on a 7-point Likert scale ranging from 1 (indicating more positive attitudes) to 7 (indicating more negative attitudes). The scale was scored by averaging all three items (α = 0.91), whereby higher scores indicate more negative attitudes towards masturbation.

##### Method of Masturbation

Participants were asked how they masturbate on four items (e.g., using fingers/hands, using sex toys, inserting fingers into the vagina/anus, and introducing some objects into the vagina/anus). They rated the frequency at which they engaged in each method using a 7-point Likert scale ranging from 1 (*never*) to 7 (*always*).

##### Pornography Consumption

Participants were asked the frequency at which they watch pornography while masturbating on a 7-point Likert scale ranging from 1 (*never*) to 7 (*all the time*). Participants were also asked why they consume pornography on a 7-point Likert scale ranging from 1 (*strongly disagree*) to 7 (*strongly agree*). From this measure, one item that was assessed in the current study is “Porn is an arousing visual aid to look at while masturbating”.

##### Reasons for Masturbation

The reasons-for-wanting-to-masturbate subscales from the Attitudes Toward Masturbation Scale (ATMS; Young & Muehlenhard, 2011) were adapted to assess people’s thoughts and reasons for engaging in masturbation with themes such as pleasure, relaxation and stress relief, and substitution for partner sex. Participants rated 13 items (e.g., “To pursue my own sexual pleasure.”) on a 7-point Likert scale ranging from 0 (*not a reason*) to 6 (*a very important reason*).

#### Frequency of Partnered Sex

Participants rated the average number of times per month they have partnered sexual activity on a 6-point Likert scale ranging from 1 (*less than once a month*) to 6 (*daily*). An option “never had sex” was provided for participants who never experienced partnered sex before.

#### Religiosity

Participants were asked in a single-item measure whether they considered themselves religious. This measure used a 7-point Likert scale ranging from 1 (*definitely not true of me*) to 7 (*definitely true of me*).

#### General Sexual Satisfaction

One item (i.e., “Over the past 4 weeks, how satisfied have you been with your overall sexual life?”) from the Female Sexual Function Inventory (FSFI; Rosen et al., 2000) was used to assess participants’ level of sexual satisfaction in the past four weeks using a 5-point Likert scale (1 = *very dissatisfied*, 5 = *very satisfied*). An option of “no sexual activity” was provided for participants to indicate if they had not experienced any sexual activity within the past four weeks. Participants who selected this option were excluded from analyses related to sexual satisfaction. Higher scores indicate higher levels of general sexual satisfaction.

#### Life Satisfaction

The Satisfaction with Life Scale (SWLS; Diener et al., 1985) was used to assess participants’ level of life satisfaction. Participants rated five items (e.g. “I am satisfied with my life.”) on a scale of 1 (*strongly disagree*) to 7 (*strongly agree*). The variable score was computed by averaging all five items’ scores (α = 0.86). Higher scores indicate higher satisfaction with life.

#### Depression

The 9-item Patient Health Questionnaire (PHQ-9; Kroenke et al., 2001) was used to assess the frequency at which participants experienced nine depressive symptoms. Participants rated these nine items (e.g., “Feeling tired or having little energy.”) on a 4-point Likert scale (0 = *not at all*, 3 = *nearly every day*). The variable score was computed by averaging all nine items’ scores (α = 0.86). Higher scores indicate higher levels of depressive symptoms.

#### Anxiety

The short version of the State-Trait Anxiety Inventory (STAI; de Vries & van Heck, 2013) was used to assess participants’ levels of trait anxiety. Participants rated ten items that assessed how they generally feel (e.g., “I feel nervous and restless.”) on a scale from 1 (*not at all*) to 4 (*very much*). Four items were reverse-scored. Item scores were averaged to form a score of anxiety (α = 0.88). Higher scores indicate greater levels of trait anxiety.

#### Stress

The 10-item Perceived Stress Scale (PSS; Cohen & Williamson, 1988; Cohen et al., 1983) was used to assess the degree to which participants found events and situations in their lives to be overwhelming, unpredictable, or uncontrollable. Participants rated ten items (e.g., “In the last month, how often have you been able to control irritations in your life?”) using a 5-point Likert scale ranging from 0 (*never*) to 4 (*very often*). Four items were reverse-scored. The scale was scored by averaging all ten items to form a stress score (α = 0.87). Higher scores indicate higher levels of perceived stress.

#### Demographics

Participants reported their gender, age, ethnicity, socioeconomic status (SES), relationship status, and religion. Subjective SES was measured using the McArthur Scale of Subjective Social Status (Adler et al., 2000) with possible scores ranging from 1 to 10, whereby higher scores indicated higher subjective SES.

## Results

### Masturbatory Behaviors

Among a total of 621 participants in our sample, 69 participants (11.1%) provided inconsistent responses related to masturbation-related items. For example, they would provide an age at which they first experienced masturbation but would declare that they had never masturbated before in the measure for masturbation frequency. 18 men and 51 women were found with such inconsistencies in their responses. After removing these inconsistent participants, we were left with 552 participants. 429 (77.7%) participants reported having masturbated at least once in their life, while 123 (22.3%) participants reported having never masturbated.

Of the 429 masturbators, 222 (51.7%) were men and 207 (48.3%) were women. Of 123 non-masturbators, 5 (4.1%) were men and 118 (95.9%) were women. Accordingly, we found that the number of men who reported having masturbated at least once in their life was significantly higher compared to women, χ(1) = 89.77, *p* < 0.001. Table 1 shows the breakdown of masturbation-related items separated by gender. The percentage of men who reported higher frequencies of masturbation was greater than women, while women reported more moderate levels of masturbation. Most men (80.1%) and women (83.0%) found masturbation to be at least somewhat enjoyable. Additionally, almost half the proportion of men (47.5%) and only 31.8% of women reported always having an orgasm during masturbation. When masturbating, a large percentage of men (37.9%) and women (28.6%) reported watching porn every time they do so. Furthermore, 37.9% of men and 36.4% of women strongly agreed that porn is an arousing visual aid for masturbation. The primary method of masturbation for both men and women appeared to be using their fingers or hands. Most men and women reported never using sex toys or inserting fingers or objects into their anus or vagina.

**Table 1:** Masturbatory behaviors among those who masturbate

Variables	Men		Women	
	*N*	%	*N*	%
*Masturbation frequency*	222	51.7	207	48.3
Less than once a month	6	2.7	41	19.8
About once a month	11	5.0	21	10.1
2–3 times per month	27	12.2	55	26.6
Once a week	30	13.5	35	16.9
Multiple times per week	106	47.7	47	22.7
Daily	27	12.2	7	3.4
Multiple times a day	15	6.8	1	0.5
*Masturbation enjoyment*	141	48.0	153	52.0
Extremely unenjoyable	0	0.0	6	3.9
Highly unenjoyable	6	4.3	4	2.6
Somewhat unenjoyable	8	5.7	6	3.9
Neither unenjoyable or enjoyable	14	9.9	10	6.5
Somewhat enjoyable	46	32.6	58	37.9
Highly enjoyable	41	29.1	48	31.4
Extremely enjoyable	26	18.4	21	13.7
*Orgasm frequency during masturbation*	202	51.3	192	48.7
Never	13	6.4	29	15.1
Rarely	5	2.5	16	8.3
Occasionally	19	9.4	31	16.1
Half the time	15	7.4	17	8.9
Frequently	26	12.9	13	6.8
Usually	28	13.9	25	13.0
Always	96	47.5	61	31.8
*Porn consumption during masturbation*	103	57.2	77	42.8
Never	1	1.0	7	9.1
Rarely	5	4.9	9	11.7
Occasionally	5	4.9	5	6.5
Sometimes	10	9.7	8	10.4
Often	12	11.7	7	9.1
Very often	31	30.1	19	24.7
All the time	39	37.9	22	28.6
*Porn as an arousing visual aid during masturbation*	103	57.2	77	42.8
Strongly disagree	0	0.0	5	6.5
Disagree	1	1.0	2	2.6
Somewhat disagree	0	0.0	2	2.6
Neither agree nor disagree	3	2.9	5	6.5
Somewhat agree	18	17.5	11	14.3
Agree	42	40.8	24	31.2
Strongly agree	39	37.9	28	36.4
*Methods of masturbation: Fingers/hands*	222	51.7	207	48.3
Never	49	22.1	30	14.5
Rarely	17	7.7	34	16.4
Occasionally	23	10.4	60	29.0
Half the time	14	6.3	18	8.7
Frequently	37	16.7	29	14.0
Usually	32	14.4	17	8.2
Always	50	22.5	19	9.2
*Methods of masturbation: Sex toys (i.e., vibrators, fleshlight *etc*.)*	222	51.7	207	48.3
Never	192	86.5	150	72.5
Rarely	15	6.8	23	11.1
Occasionally	7	3.2	16	7.7
Half the time	4	1.8	1	0.5
Frequently	2	0.9	5	2.4
Usually	2	0.9	6	2.9
Always	0	0.0	6	2.9
*Methods of masturbation: Inserting fingers into vagina/anus*	222	51.7	207	48.3
Never	188	84.7	93	44.9
Rarely	19	8.6	56	27.1
Occasionally	4	1.8	32	15.5
Half the time	7	3.2	8	3.9
Frequently	0	0.0	8	3.9
Usually	0	0.0	7	3.4
Always	4	1.8	3	1.4
*Methods of masturbation: Introducing some objects into the vagina/anus*	222	51.7	207	48.3
Never	198	89.2	167	80.7
Rarely	15	6.8	25	12.1
Occasionally	4	1.8	9	4.3
Half the time	3	1.4	2	1.0
Frequently	0	0.0	1	0.5
Usually	1	0.5	3	1.4
Always	1	0.5	0	0.0

Compared to women (*M* = 14.7 years, *SD* = 3.8 years), men (*M* = 13.1 years, *SD* = 2.4 years) reported first masturbating at a significantly younger age (*t* = -5.13, *p* < 0.001). Men (*M* = 3.3, *SD* = 1.5) also reported significantly more positive attitudes towards masturbation (*t* = 2.05, *p* = 0.04) as compared to women (*M* = 2.9, *SD* = 1.3). With regards to the time taken for participants to orgasm when masturbating, men (*M* = 14.2 min, *SD* = 11.0 min) generally did not take significantly longer time (*t* = 1.69, *p* = 0.09) than women (*M* = 12.2, *SD* = 12.6), however there was high variability in orgasm latency for both genders. In terms of the reasons both men and women engage in masturbation, the top five that were rated as very important to moderately important reasons were “to feel sexually satisfied”, “to pursue my own sexual pleasure”, “to have an orgasm”, “to relieve stress”, and “to sleep better”.

### Differences Between Masturbators and Non-Masturbators

Independent-samples T-tests were run to examine the difference in sexual and psychological well-being between individuals who reported having masturbated before and those who reported having never masturbated in their life. As summarized in Table S1, sexual satisfaction and psychological well-being did not vary significantly between masturbators and non-masturbators (*ps* > 0.25).

### Associations Between Masturbation Frequency and Well-Being Among Masturbators

Descriptive statistics and bivariate correlations between all variables of interest for the subsample of individuals with masturbation experience are summarized in Table S2. To examine whether masturbation frequency was associated with sexual and psychological well-being, hierarchical linear regressions were computed. Phase of study, age, and SES were tested as control variables in Step 1, masturbation frequency and moderator variables (i.e., gender, frequency of partnered sex, partner availability, and religiosity) were entered in Step 2, and the two-way interaction terms between masturbation frequency and each moderator were entered in Step 3.

As summarized in Table 2, results revealed that the addition of the two-way interaction terms did not significantly improve the model for all outcome variables, *p*s > 0.25. Gender, frequency of partnered sex, availability of partner, and religiosity did not moderate the association between masturbation frequency and outcome variables. Thus, results from Step 2, which excluded the interaction terms, were interpreted.

**Table 2:** Hierarchical linear regressions predicting sexual satisfaction and psychological well-being from masturbation frequency and potential moderators

Outcomes	General sexual satisfaction	Life satisfaction	Depression	Anxiety	Stress
*Step 1: Covariates*					
Phase of study	− 0.11	− 0.15	0.07	0.08	0.19**
Age	0.06*	0.05	− 0.03*	− 0.05***	− 0.05***
SES	− 0.01	0.29***	− 0.09***	− 0.08***	− 0.09***
*R*^*2*^	0.01	0.11	0.06	0.07	0.08
*F*	1.58	16.56***	8.32***	10.12***	11.62***
*Step 2: Masturbation frequency and proposed moderators*					
Phase of study	0.03	− 0.12	0.10	0.10	0.20**
Age	0.01	0.03	− 0.03*	− 0.05***	− 0.06***
SES	− 0.03	0.28***	− 0.09***	− 0.08***	− 0.09***
Masturbation frequency	− 0.11**	− 0.05	0.04	0.07**	0.05*
Gender	− 0.06	− 0.13	0.26***	0.36***	0.25***
Frequency of sex	0.16***	− 0.01	0.04	0.01	0.003
Partner availability	0.70***	0.38**	− 0.19**	− 0.08	0.02
Religiosity	0.04	0.09*	0.01	− 0.01	− 0.03
*R*^*2*^	0.21	0.14	0.11	0.14	0.12
Δ*R*^*2*^	0.20	0.04	0.05	0.07	0.04
Δ*F*	19.91***	3.42**	4.63***	6.70***	3.62**
*Step 3: Interaction terms*					
Phase of study	0.03	− 0.12	0.10	0.10	0.20**
Age	0.01	0.03	− 0.03*	− 0.05***	− 0.06***
SES	− 0.03	0.27***	− 0.09***	− 0.08***	− 0.09***
Masturbation frequency	− 0.10	0.08	0.02	0.09	0.06
Gender	0.21	0.13	0.28	0.38	0.12
Frequency of sex	0.23	0.23	0.03	− 0.02	0.02
Partner availability	0.05	− 0.09	− 0.35	0.07	0.12
Religiosity	0.07	0.23	0.001	− 0.001	− 0.004
Gender × Masturbation frequency	− 0.05	− 0.05	− 0.003	− 0.01	0.03
Frequency of sex × Masturbation frequency	− 0.01	− 0.04	0.001	0.01	− 0.003
Partner availability × Masturbation frequency	0.13	0.09	0.03	− 0.03	− 0.02
Religiosity × Masturbation frequency	− 0.01	− 0.03	0.002	− 0.002	− 0.004
*R*^*2*^	0.22	0.15	0.11	0.14	0.12
Δ*R*^*2*^	0.01	0.01	0.002	0.001	0.002
Δ*F*	0.74	1.01	0.21	0.18	0.20

Phase of study was dummy coded as 0 = phase 1 and 1 = phase 2, gender as 0 = male and 1 = female, and availability of partner as 0 = had an available partner and 1 = no available partner

*p < .05, **p < .01, ***p < .001

Controlling for the phase of study, age, SES, gender, frequency of partnered sex, availability of partner, and religiosity, masturbation frequency was found to be significantly associated with lower general sexual satisfaction, higher trait anxiety, and higher stress. Life satisfaction and depression did not vary as a function of masturbation frequency. 

Other significant correlates of sexual satisfaction include having a partner and more frequent partnered sex. Having a partner, higher subjective SES, and being more religious were significantly associated with higher satisfaction with life. Additionally, other significant correlates of depressive symptoms include being female, younger in age, not having an available sexual partner, and having lower subjective SES. Being a woman, younger in age, and having lower subjective SES were also significantly associated with higher trait anxiety. Furthermore, women, younger participants, those in the second phase of the study, and those with lower subjective SES were found to have significantly higher stress levels.

## Discussion

The current study examined the masturbatory behaviors of a sample of Malaysian young adults and the association between masturbation frequency with sexual and psychological well-being. We hypothesized that participants who reported having masturbated before would have significantly higher levels of sexual satisfaction, depression, and anxiety but significantly lower levels of life satisfaction and stress compared to participants who reported having never masturbated before. Our findings do not support our hypothesis as we found no differences in general sexual satisfaction and psychological well-being between masturbators and non-masturbators. We also expected masturbation frequency to be associated with sexual and psychological well-being among masturbators, and that these associations would vary by gender, frequency of partnered sex, availability of a sexual partner, and religiosity. Findings indicated that masturbation frequency was positively associated with stress and anxiety and negatively associated with sexual satisfaction. Contrary to our hypotheses, these associations were consistent across gender, frequency of partnered sex, partner availability, and religiosity. Masturbation frequency was not associated with depression or satisfaction with life.

In the current study, 77.7% of participants reported masturbating at least once in their life, which is higher than the prevalence of 28.5% reported in previous research conducted on Malaysian late adolescents (Awaluddin et al., 2015) but slightly lower than the 76.8% prevalence reported among German young adults (Velten & Margraf, 2017). This may be due to our reliance on convenience sampling or our larger age range of 18 − 30 years compared to Awaluddin et al.’s age range of 18 − 19 years. Specifically, 97.8% of men and 63.7% of women in our sample (after removing inconsistent participants) reported having masturbated at least once in their life, which is a higher percentage of men (85.5%) and a lower percentage of women (68.3%) compared to German young adults (Velten & Margraf, 2017). In line with past findings (Pinkerton et al., 2003), we found that men reported masturbating earlier in their life and more frequently than women.

Most masturbators reported enjoying masturbating and engaging in masturbation multiple times per week. This is consistent with findings conducted with German participants (Burri & Carvalheira, 2019; Velten & Margraf, 2017) and Chinese participants who report an average masturbation frequency of six times per month (Jiao et al., 2019). Consistent with Miller et al.’s (2019) findings, we found that most men (79.7%) and women (62.4%) often, very often or always consume porn during masturbation. As for orgasm functioning, only 47.5% of men and 31.8% of women reported always achieving orgasm during masturbation, which is relatively low compared to existing studies that reported rates of over 90.0% for men (Dekker & Schmidt, 2003) and 50.3% for women (Burri & Carvalheira, 2019). This could be attributed to the lower variety (Fugl-Meyer et al., 2006) of masturbatory behaviors observed in the current sample, most of which reported having never masturbated using sex toys or penetrating their genitals with fingers or objects. This may reflect lower openness to sex. Indeed, orgasm during masturbation tends to be lower among those with less sexually permissive attitudes and more sexual shame (Lentz & Zaikman, 2021).

Alternatively, the lower orgasm frequency during masturbation reported in our sample could be due to differences in reasons for masturbation. However, our findings showed that the top five reasons for masturbation endorsed in the current study were “to feel sexually satisfied”, “to have an orgasm”, “to pursue my own sexual pleasure”, “to sleep better”, and “to relieve stress”, which are similar to findings derived from a sample of German women (Burri & Carvalheira, 2019). Given these consistent motivations for masturbation across both samples, it is unlikely that differences in masturbation motivation contribute to the lower orgasm frequency during masturbation in our study.

In contradiction to past findings on the superior sexual satisfaction of those who masturbate compared to those who do not (Hurlbert & Whittaker, 1991), we found that general sexual satisfaction, life satisfaction, depression, stress, and anxiety did not vary between masturbators and non-masturbators. Among the masturbators, however, people who reported masturbating more often tended to report being less satisfied with their overall sex life. This finding does not support the argument that masturbation can increase general sexual satisfaction through the promotion of sexual self-efficacy and autonomous self-provision of sexual pleasure (Bowman, 2014). Rather, it is aligned with studies highlighting the importance of partnered sex in achieving sexual satisfaction (Brody & Costa, 2009). Indeed, we found that more frequent partnered sex significantly predicted higher general sexual satisfaction. The negative association between masturbation frequency and sexual satisfaction was not conditional on gender, partnered sex frequency, partner availability or religiosity, which further highlights the robustness of this association.

Results from the current study also suggested that people who reported masturbating more often tended to report more symptoms of stress and anxiety. This is consistent with past research that found positive associations between masturbation and anxiety/depression (Rowland et al., 2020), with the exception that our study did not find a significant relationship between masturbation frequency and depression. However, Rowland et al. (2020) measured anxiety/depression using one dichotomous item, while our study utilized more comprehensive measures of depression and anxiety (i.e., the PHQ-9 and STAI respectively). Anxiety and depression have been investigated concurrently as either predictors or outcomes of sexual behaviors or functioning (Kalmbach et al., 2014; Rowland et al., 2020). However, our results illustrate that masturbation may be specifically associated with anxiety and not depression, albeit being related constructs. A likely explanation for this is that people use masturbation as a means to manage their anxiety and distress (Fahs & Frank, 2014). Perhaps masturbation can also be argued to be a short-term coping mechanism for anxiety and stress rather than a solution to improve general mental health. Apart from masturbation as a coping mechanism, it could be possible that the association between more frequent masturbation with higher levels of anxiety and stress in our sample is attributed to the shame that accompanies the behavior.

Nonetheless, our findings revealed that gender, frequency of partnered sex, partnered availability, and religiosity did not moderate the associations between masturbation with sexual and psychological well-being outcomes. From a biological perspective, both men and women experience similar brain responses for the orgasmic phase (Georgiadis et al., 2009). Thus, it is justifiable that the outcome of masturbation would be similar for both genders from biological and psychological perspectives. Additionally, it may be that partnered sexual activity may not have an impact on the relationship between self-stimulation and well-being among our sample, as the second phase of data collection was conducted amid a movement restriction order due to the COVID-19 pandemic. Furthermore, our sample might hold more liberal values as religiosity was positively skewed, reflecting low religiosity in our sample.

### Limitations and Suggestions for Future Research

Although the current study provided insights into the masturbatory practices of a sample of Malaysian young adults, the generalizability of our findings may be lacking due to our reliance on convenience sampling. In terms of sample proportion, the majority of participants in the current sample were of Chinese ethnicity (58.8%), which is not representative of the ethnicity distribution (Malays make up the majority with 57.8% as of 2022) of young adults in Malaysia (Department of Statistics Malaysia, 2022). Data was collected using a self-administered questionnaire that may lend itself to social desirability bias, considering the sensitive nature of the questions asked regarding participants’ sexual behaviors and motivations. However, we note that our findings revealed similar patterns of masturbatory behaviors and attitudes with past research conducted with German and Chinese samples. Additionally, some participants were inconsistent in their responses to various masturbation-related items. While these participants were excluded from our analyses, it is worth noting that this inconsistent reporting was observed more frequently among women than men. This may be due to participants’ general inexperience towards masturbation or unfamiliarity with the wording used to describe various masturbatory behaviors. It may also reflect a sense of shame in reporting masturbatory behaviors, as reflected in the negative view of masturbation in Malaysian society (Low et al., 2007; Sidi et al., 2013).

Furthermore, given that the current study was part of a larger research project, our variables of interest, particularly sexuality-related, were mostly captured by single-item measures or brief versions of validated scales. It is possible that some of our measures were unable to fully capture specific dimensions of our constructs and their associations with each other. It would be worthwhile to utilize more sophisticated measures of masturbation and sexual well-being in future studies.

Moreover, the cross-sectional nature of our study precludes causal inference. For instance, we cannot conclude with certainty that it is masturbation that predicts sexual satisfaction and psychological well-being. It is possible that people who are more satisfied with their sex life are less motivated to masturbate, given that their sexual needs are already satisfied (Regnerus et al., 2017). We conducted post-hoc analyses to investigate general sexual satisfaction, life satisfaction, depression, stress, and anxiety as predictors of masturbation frequency (see Table S3). Similar to our main findings, analyses showed that lower general sexual satisfaction, higher stress and anxiety levels were significantly associated with higher frequency of masturbation, while life satisfaction and depression were not significant predictors of masturbation frequency. Although the current study operates on a different definition of sexual satisfaction, Regnerus et al. (2017) found that subjective contentment with the recent frequency of sex was a good predictor of masturbation frequency. Furthermore, much research has suggested that overcoming anxious feelings and reducing stress are common motivations for masturbation (Csako et al., 2022; Kalmbach et al., 2014; Rowland et al., 2020). Thus, our findings suggest that while there were significant associations between masturbation with general sexual satisfaction, anxiety, and stress, conclusions regarding the directionality of these relationships should not be drawn at this stage. To improve confidence in making causal inferences, future studies should consider a longitudinal design to examine masturbation practices and their associated factors and outcomes over time.

### Implications

It is notable that in Malaysia, a predominantly Islamic country, the stigma against masturbation still persists. Despite being exploratory and descriptive, this study adds to the understanding of masturbation practices among Malaysian young adults. It outlines that future research should employ in-depth investigations, such as interviews, to expand upon knowledge regarding the reasons, attitudes, and perceptions of masturbation in Malaysia. Thereafter, a culturally fit intervention could be designed to promote healthy sexual and psychological outcomes through solo sexual practices, focusing on the existing negative public attitudes about solo sex and the destigmatization of masturbation among Malaysian young adults. Furthermore, the associations found in our study were not varied by our chosen moderators, which could reflect a more consistent evaluation of masturbatory practices in a culturally conservative culture. Accordingly, the non-significant association between masturbation frequency and life satisfaction further suggests that self-pleasure may have less importance in the overall well-being of Malaysian young adults.

In addition to contributing to the research on the correlates of masturbation, our study provides a culturally different lens of looking into the masturbatory habits of young adults. Furthermore, the negative association found between masturbation frequency and general sexual satisfaction suggests that perhaps interventions to boost sexual satisfaction among Malaysian young adults may require more effort than simply encouraging self-stimulation, particularly by reducing shame towards sexuality in general. Comprehensive sex education that equips young adults with accurate information on sexual health (Leiblum & Wiegel, 2002) and helps them develop positive attitudes towards sexuality (Volk et al., 2016) may aid in reducing shame related to their sexuality. Additionally, having educators facilitate and encourage students to engage in non-judgemental discussions about masturbation may further reduce the stigma of talking about sexuality and sexual behaviors, increasing a sense of autonomy which promotes more positive attitudes towards sex (Emmerink et al., 2016). Furthermore, our findings imply that having an available sexual partner and more partnered sex increases general sexual satisfaction. It follows that additional education and resources on initiating and maintaining sexual or romantic relationships may also improve the likelihood of increasing sexual satisfaction among Malaysian young adults.

## Conclusion

All in all, the current research is the first exploratory study looking into the masturbation practices of a sample of Malaysian young adults. Our findings indicated that masturbation is a commonly practiced sexual activity, as most participants reported masturbating at least once in their lifetime. However, contrary to the expected benefits of masturbation, neither sexual nor psychological well-being was significantly higher among masturbators compared to non-masturbators. In addition, higher frequency of masturbation was associated with lower general sexual satisfaction as well as higher levels of stress and anxiety. These findings suggest that cultural context should be examined alongside masturbatory behaviors so as to take into account the societal factors that encompass individuals and their expressions of sexuality.

## Supplementary Information

Below is the link to the electronic supplementary material.

Supplementary file 1 (DOCX 22 KB)
