# Supplementary material for: Masturbation Among Malaysian Young Adults: Associated Sexual and Psychological Well-Being Outcomes
Source: Sex Cult. 2023 May 26:1–21. Online ahead of print. doi: 10.1007/s12119-023-10101-2 (PMC10213586; doi:10.1007/s12119-023-10101-2)
Supplement: Supplementary file 1 — (DOCX 22 KB) [file 12119_2023_10101_MOESM1_ESM.docx]

**Supplementary Tables**

**Table S1.** General sexual satisfaction and psychological well-being outcomes between masturbators and non-masturbators

| **Outcome variables** | **Masturbators** | **Non-masturbators** | **T-statistic** | ***p*** |
| --- | --- | --- | --- | --- |
|  | ***M* (*SD*)** | ***M* (*SD*)** |  |  |
| General sexual satisfaction | 3.3 (1.2) | 3.3 (1.2) | 0.22 | .83 |
| Life satisfaction | 3.9 (1.4) | 3.9 (1.3) | 0.20 | .84 |
| Depression | 1.0 (0.6) | 0.9 (0.6) | -0.48 | .63 |
| Anxiety | 2.4 (0.6) | 2.5 (0.7) | 0.72 | .47 |
| Stress | 2.0 (0.7) | 2.1 (0.7) | 1.14 | .26 |

**Table S2.** Zero-order correlations between variables of interest

| **Variables** | **1.** | **2.** | **3.** | **4.** | **5.** | **6.** | **7.** | **8.** | **9.** | **10.** | **11.** | **12.** | **13.** |
| --- | --- | --- | --- | --- | --- | --- | --- | --- | --- | --- | --- | --- | --- |
| 1. Phase of study | 1 |  |  |  |  |  |  |  |  |  |  |  |  |
| 2. Age | .38*** | 1 |  |  |  |  |  |  |  |  |  |  |  |
| 3. SES | -.05 | .04 | 1 |  |  |  |  |  |  |  |  |  |  |
| 4. Gender | -.09 | .005 | .04 | 1 |  |  |  |  |  |  |  |  |  |
| 5. Frequency of sex | -.03 | .14** | .05 | .03 | 1 |  |  |  |  |  |  |  |  |
| 6. Partner availability | .03 | .18*** | .02 | .14** | .43*** | 1 |  |  |  |  |  |  |  |
| 7. Religiosity | -.04 | .002 | .05 | .02 | -.20*** | -.04 | 1 |  |  |  |  |  |  |
| 8. Masturbation frequency | .08 | .03 | -.03 | -.44*** | .09 | -.08 | -.15** | 1 |  |  |  |  |  |
| 9. Sexual satisfaction | .002 | .10* | -.007 | .09 | .34*** | .40*** | .01 | -.14** | 1 |  |  |  |  |
| 10. Life satisfaction | -.04 | .08 | .32*** | .02 | .04 | .14** | .14** | -.06 | .16** | 1 |  |  |  |
| 11. Depression | .02 | -.12* | -.21*** | .13** | .02 | -.10* | -.004 | .02 | -.07 | -.48*** | 1 |  |  |
| 12. Anxiety | .004 | -.18*** | -.19*** | .18*** | .003 | -.05 | -.07 | .05 | -.12* | -.57*** | .66*** | 1 |  |
| 13. Stress | .09 | -.14** | -.21*** | .11* | .006 | .005 | -.10* | .06 | -.12* | -.55*** | .66*** | .77*** | 1 |
| *M* | - | 22.01 | 6.29 | - | 1.21 | - | 3.33 | 4.96 | 3.31 | 3.88 | 0.96 | 2.43 | 2.03 |
| *SD* | - | 2.38 | 1.45 | - | 1.71 | - | 1.81 | 1.58 | 1.21 | 1.36 | 0.65 | 0.64 | 0.65 |
| *N* | 429 | 427 | 428 | 429 | 429 | 427 | 429 | 429 | 407 | 429 | 429 | 429 | 429 |

*Note*. Phase of study was dummy coded as 0 = phase 1 and 1 = phase 2, gender as 0 = male and 1 = female, and availability of partner as 0 = had an available partner and 1 = no available partner. **p* < .05, ***p* < .01, ****p* < .001.

**Table S3.** Hierarchical linear regressions predicting masturbation frequency from general sexual satisfaction, psychological well-being and potential moderators

| **Predictors** | **General sexual satisfaction** | **Life satisfaction** | **Depression** | **Anxiety** | **Stress** |
| --- | --- | --- | --- | --- | --- |
| **Step 1: Covariates** |  |  |  |  |  |
| Phase of study | 0.46** | 0.26 | 0.26 | 0.26 | 0.26 |
| Age | -0.01 | 0.003 | 0.003 | 0.003 | 0.003 |
| SES | -0.04 | -0.03 | -0.03 | -0.03 | -0.03 |
| *R^2^* | 0.02 | 0.01 | 0.01 | 0.01 | 0.01 |
| *F* | 2.92* | 1.11 | 1.11 | 1.11 | 1.11 |
|  |  |  |  |  |  |
| **Step 2: Predictors and proposed moderators** |  |  |  |  |  |
| Phase of study | 0.27 | 0.10 | 0.09 | 0.07 | 0.06 |
| Age | 0.01 | 0.02 | 0.02 | 0.03 | 0.03 |
| SES | -0.02 | 0.004 | 0.01 | 0.02 | 0.01 |
| Predictor | -0.17** | -0.06 | 0.20 | 0.38** | 0.27* |
| Gender | -1.30*** | -1.35*** | -1.39*** | -1.44*** | -1.39*** |
| Frequency of sex | 0.11* | 0.10* | 0.09* | 0.10* | 0.10* |
| Partner availability | -0.12 | -0.22 | -0.20 | -0.21 | -0.24 |
| Religiosity | -0.09* | -0.10* | -0.11** | -0.10* | -0.10* |
| *R^2^* | 0.24 | 0.23 | 0.23 | 0.25 | 0.24 |
| Δ*R^2^* | 0.22 | 0.22 | 0.22 | 0.24 | 0.23 |
| Δ*F* | 23.18*** | 23.72*** | 24.23*** | 26.20*** | 24.87*** |
|  |  |  |  |  |  |
| **Step 3: Interaction terms** |  |  |  |  |  |
| Phase of study | 0.27 | 0.12 | 0.10 | 0.07 | 0.06 |
| Age | 0.01 | 0.01 | 0.02 | 0.03 | 0.03 |
| SES | -0.02 | 0.01 | 0.01 | 0.02 | 0.01 |
| Predictor | -0.05 | 0.11 | -0.13 | 0.26 | 0.14 |
| Gender | -0.95* | -1.00* | -1.43*** | -1.37* | -1.78*** |
| Frequency of sex | 0.16 | 0.26 | 0.06 | -0.04 | 0.07 |
| Partner availability | -0.37 | -0.60 | -0.48 | 0.09 | -0.03 |
| Religiosity | -0.02 | 0.05 | -0.15* | -0.18 | -0.13 |
| Gender x Predictor | -0.11 | -0.09 | 0.05 | -0.03 | 0.19 |
| Frequency of sex x Predictor | -0.02 | -0.04 | 0.04 | 0.06 | 0.01 |
| Partner availability x Predictor | 0.08 | 0.10 | 0.30 | -0.12 | -0.10 |
| Religiosity x Predictor | -0.02 | -0.04 | 0.04 | 0.03 | 0.02 |
| *R^2^* | 0.25 | 0.24 | 0.24 | 0.25 | 0.24 |
| Δ*R^2^* | 0.003 | 0.01 | 0.01 | 0.002 | 0.002 |
| Δ*F* | 0.41 | 0.86 | 0.75 | 0.21 | 0.26 |

*Note.* Masturbation frequency is the outcome variable for all five models of regression. * *p* < .05, ** *p* < .01, *** *p* < .001
